# Markovian Analysis of the Sequential Behavior of the Spontaneous Spinal Cord Dorsum Potentials Induced by Acute Nociceptive Stimulation in the Anesthetized Cat

**DOI:** 10.3389/fncom.2017.00032

**Published:** 2017-05-01

**Authors:** Mario Martin, Javier Béjar, Gennaro Esposito, Diógenes Chávez, Enrique Contreras-Hernández, Silvio Glusman, Ulises Cortés, Pablo Rudomín

**Affiliations:** ^1^Universitat Politècnica de Catalunya (BarcelonaTech)Barcelona, Spain; ^2^Department of Physiology, Biophysics and Neurosciences, Center for Research and Advanced Studies, National Polytechnic InstituteMexico City, Mexico; ^3^Stroger Cook County HospitalChicago, IL, USA; ^4^Barcelona Supercomputing CenterBarcelona, Spain; ^5^El Colegio NacionalMexico City, Mexico

**Keywords:** machine learning, markovian analysis, nociceptive stimulation, cord dorsum potentials, spinal cord

## Abstract

In a previous study we developed a Machine Learning procedure for the automatic identification and classification of spontaneous cord dorsum potentials (*CDPs*). This study further supported the proposal that in the anesthetized cat, the spontaneous *CDPs* recorded from different lumbar spinal segments are generated by a distributed network of dorsal horn neurons with structured (non-random) patterns of functional connectivity and that these configurations can be changed to other non-random and stable configurations after the noceptive stimulation produced by the intradermic injection of capsaicin in the anesthetized cat. Here we present a study showing that the sequence of identified forms of the spontaneous *CDPs* follows a Markov chain of at least order one. That is, the system has memory in the sense that the spontaneous activation of dorsal horn neuronal ensembles producing the *CDPs* is not independent of the most recent activity. We used this markovian property to build a procedure to identify portions of signals as belonging to a specific functional state of connectivity among the neuronal networks involved in the generation of the *CDPs*. We have tested this procedure during acute nociceptive stimulation produced by the intradermic injection of capsaicin in intact as well as spinalized preparations. Altogether, our results indicate that *CDP* sequences cannot be generated by a renewal stochastic process. Moreover, it is possible to describe some functional features of activity in the cord dorsum by modeling the *CDP* sequences as generated by a Markov order one stochastic process. Finally, these Markov models make possible to determine the functional state which produced a *CDP* sequence. The proposed identification procedures appear to be useful for the analysis of the sequential behavior of the ongoing *CDPs* recorded from different spinal segments in response to a variety of experimental procedures including the changes produced by acute nociceptive stimulation. They are envisaged as a useful tool to examine alterations of the patterns of functional connectivity between dorsal horn neurons under normal and different pathological conditions, an issue of potential clinical concern.

## 1. Introduction to the problem

Previous work (Manjarrez et al., 2000, 2003; Chávez et al., 2012) has indicated that some specific classes of spontaneous cord dorsum potentials (*CDPs*) appear specially associated with the activation of spinal pathways that lead to primary afferent depolarization and presynaptic inhibition. These and other studies led to the proposal that in the anesthetized cat these *CDPs* are generated by a segmentally distributed network of dorsal horn neurons with structured (non-random) patterns of functional connectivity between them, and that these patterns can be changed to other, also non-random and stable configurations, after the activation of nociceptive pathways induced by the intradermic injection of capsaicin.

Quite recently, we developed a Machine Learning procedure that allows the automatic selection and classification of *CDPs* (Martín et al., 2015). We used this procedure allowed us to automate the selection and systematic analysis of the spontaneous *CDPs* recorded along several hours of continuous recording. This revealed emerging non-random classes of *CDPs* that could be identified and compared among them. The resulting configurations behaved in a similar way across different experiments.

We now use this feature to build *dictionaries* of *CDPs*. That is, of a repertoire of specific classes of *CDPs* produced by neuronal activity, that allow us to discretize the recorded data as a sequence of symbols. We use a Markovian approach to determine (a) the extent to which different sequences of *CDPs* obtained from the same experiment are similar and (b) the dependence of spinal changes induced by nociception on supraspinal modulatory influences (see Section 6). To this end, we analyzed raw data from already published studies performed in anesthetized cats. We found that in both cases, those ensembles generate emergent phases of activity that are identifiable and follow a Markov chain of order one. That is, the system has memory in the sense that firing of neurons is not independent of the preceding neuronal activation. In view of the concurrent inhibition that follows individual *CDPs* (Contreras-Hernández et al., 2015), it is suggested that the statistical dependence between successive *CDPs* results, at least in part, from a structured (non-random) activation of the inhibitory GABAergic and glycinergic pathways that follow the generation of particular classes of *CDPs*.

In addition, we use this markovian property to build a procedure to identify portions of signals as belonging to a specific functional state of the connectivity between the neuronal networks involved in the generation of particular classes of *CDPs*. This identification procedure appears to be useful for the analysis of the sequential behavior of the ongoing *CDPs* recorded from different spinal segments in response to a variety of experimental procedures, among them, the changes produced by acute nociceptive stimulation induced by the intradermic injection of capsaicin.

Results we present in this paper indicate that: 1. *CDPs* sequences cannot be generated by a renewal (memoryless, or Markov order zero) stochastic process. 2. By modeling the *CDPs* sequences as generated by a Markov order one stochastic process it is possible to describe some functional features of the activity of the dorsal horn neurons involved in the generation of the *CDPs*$\dot{3}$. Based on the transition matrix calculated for experimental sequence it is possible to determine the functional state which produced that sequence. These procedures are envisaged as a useful tool to examine alterations in the time domain of the patterns of functional connectivity between dorsal horn neurons under normal and different pathological conditions, an issue of potential clinical concern.

### 1.1. Outline of the paper

Section 2 describes the procedure for the acquisition of the signals and the experimental setup. It includes the definition of the set of experimental procedures performed in anesthetized cats. In Section 3 we detail the methodology used to extract the symbols corresponding to the sequence's elements of the recorded signals. Section 4 deals with the probabilistic modeling to describe the appearance of different symbols in a raw recording. From this modeling we can see that each experimental maneuver shows a different probabilistic distribution in pairs of consecutive symbols.

In Section 5 we prove, using statistical methods, that the sequence of symbols is markovian of order one. Section 6 shows how to make profit of this markovian property to identify to which among several experimental maneuvers a given sequence of data belongs. We also show the performance of this method using data from available experiments. Finally, Section 7 provides some conclusions and future directions of this work.

## 2. Data acquisition

In this work we used data obtained from experiments performed in anesthetized cats, paralyzed, and maintained under artificial ventilation (see Chávez et al., 2012 for a general description of the experimental procedures). Briefly, spontaneous *CDPs* were recorded by means of a matrix of silver ball electrodes (from 8 to 12 depending on the experiment) placed on the cord dorsum in both sides of the *L*4–*L*7 spinal segments against a similar number of indifferent electrodes, each inserted on the paravertebral muscles close to the active electrode using AC amplifiers with filters set from 0.3 Hz to 10 kHz.

The experiments lasted between 6 and 10 h and the signals were recorded and stored digitally for off-line analysis. The recordings made during the experiments are divided in different steps, each one of them composed by a number of independent *time steps* (*s*_1_…*s*_*j*_) of equal time duration (e.g., divided in sets of lasting 10 minutes of continuous recording).

The aim is to detect the changes of the patterns of neuronal activity in the dorsal horn produced by the different maneuvers performed during the experiment. All the studied experiments record the activity for non contiguous periods where it is assumed that evident changes would appear.

The initial step of the experiments includes the spontaneous *CDPs* recorded under control conditions during a 30–60 min period. It is referred as control period (*ctrl*). It measures the events in the dorsal horn neurons of the cat spinal cord in the absence of purposeful stimulation. Data are registered for a number of successive time steps before performing a specific procedure.

In this set of experiments we also examined changes in spontaneous *CDPs* induced by nociceptive stimulation produced by the intradermic injection of capsaicin into the left footpad in preparations with intact neuroaxis. It is referred as capsaicin period (*capsa*). The injection of capsaicin activates the nociceptive peripheral receptors that send signals mostly to the dorsal horn neurons located in the *L*5 and *L*6 lumbar segments. This period has a duration of 3–4 h. Some of the data presently analyzed were obtained from experiments aimed to examine the effects of capsaicin injected after acute spinalization in order to eliminate supraspinal descending influences in the generation of the spontaneous *CDPs*. For this purpose the spinal cord was cooled and sectioned at T10 level. This maneuver is referred as spinalization period (*esp*).

Table 1 shows the experiments presently analyzed and the procedures followed in each of them. In the text, the different procedures are labeled as *ctrl*, *capsa*, and *esp*, and the numbers that follow refer to the order in which they were recorded. For example, *ctrl*_2_ refers to the second time step recording of 10 min during the control period of the experiment.

**Table 1:** **Maneuvers performed in the experiments presently analyzed and the number of time steps**.

**Experiment**	**Maneuvers (number of steps)**
e110906	*ctrl* (2), *capsa* (3)
e120511	*ctrl* (2), *capsa* (4), *esp* (1)
e130221	*ctrl* (2), *esp* (2), *capsa* (4)
e140225	*ctrl* (2), *esp* (5), *capsa* (5)

*All time steps have a duration of 10 min each. See text for further explanations*.

Through all the paper the experiment e130221 will be used as running example to illustrate the different steps of the methodology and its results. In Section 6.1 the results from the other experiments will be discussed.

## 3. Sequence extraction

**Figure 1:**
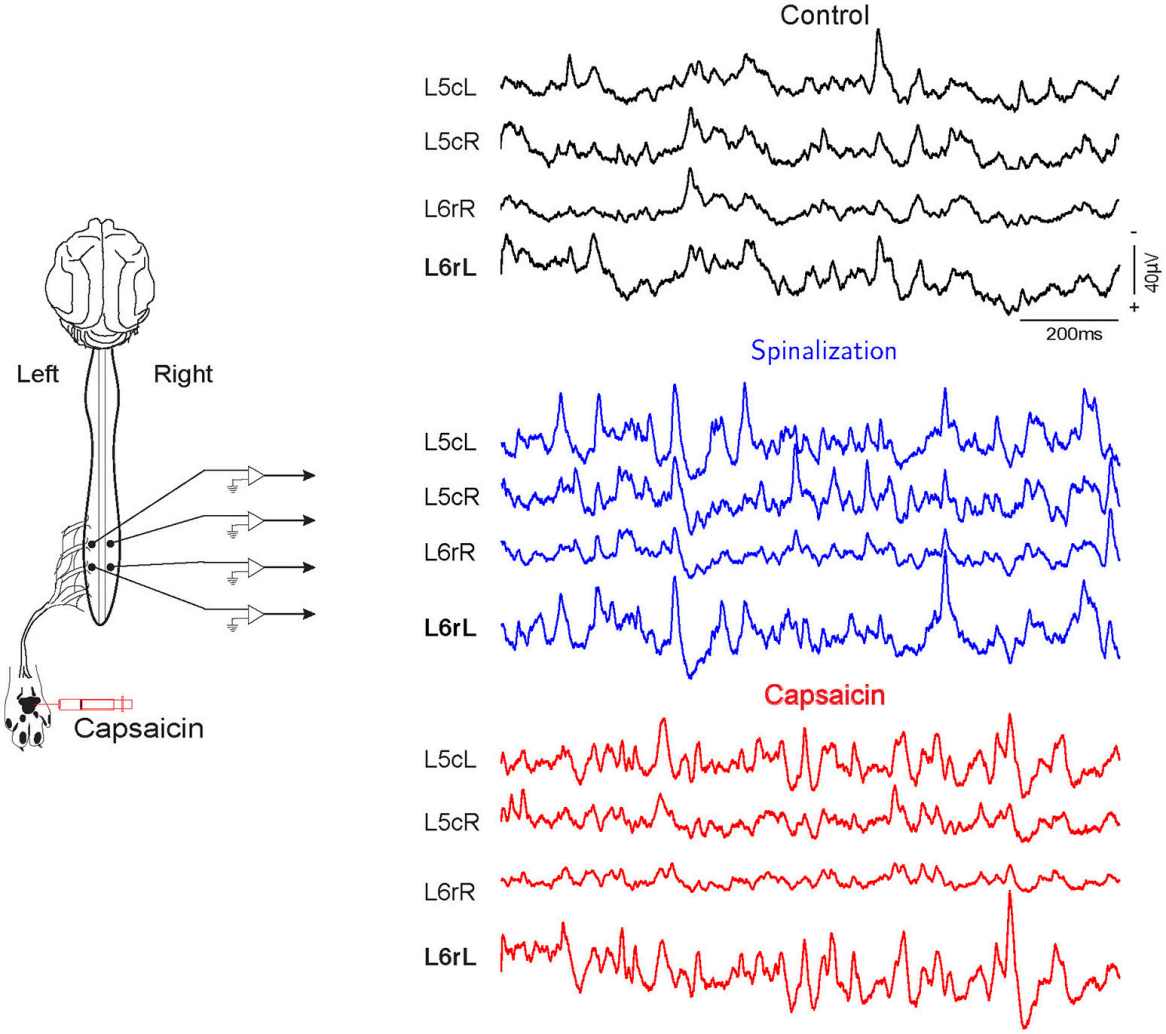
**Spontaneous *CDPs* recorded from L5 to L6 lumbar segments during control, after spinalization and 60 min after the subsequent intradermic injection of capsaicin**. Note that the *CDPs* increased after spinalization and that capsaicin had different effect on the *CDPs* recorded in the left and right side of the spinal cord. L, left; R, right; c caudal; r, rostral. Data obtained from experiment e130221 (negative voltages plotted upward).

Simultaneous recordings from the cord dorsum reveal spontaneous potentials of different shapes and amplitudes, as shown in Figure 1. The amplitudes and distributions of these potentials are quite variable, but specific patterns of *CDPs* simultaneously generated in different spinal segments are also observable during a given time step (see Chávez et al., 2012).

In order to extract a sequence of *CDPs* from the recordings, we use the methodology for the analysis of populations of neuronal signals described in Martín et al. (2015) and Béjar et al. (2015). This methodology comprises several phases, from the extraction of the events in the signal to the analysis of the events behavior at different levels of granularity in the spatial and temporal dimensions.

The first three phases of the methodology aim to build *dictionaries of the events* relevant for the analysis. In our domain, the relevant events are the *CDPs*. As an initial step, we apply an automated and unsupervised *CDPs* detection method assuming smoothness in the definition of the *CDPs* candidates and considering that they appear as peaks in the signal. It is assumed that the background noise in the recordings is stationary, essentially Gaussian and also independent from the neuronal signals. Under these assumptions, an automatic event extraction algorithm detects peaks on the signal using a sliding window large enough to contain the events. The signal inside the window is smoothed and selected as *CDP* candidate if there is a peak at its center and holds some shape constraints. The smoothing is used only to detect the position of the maximum of the window, and it also allows to maximize the ratio noise signal so it is minimized the amount of spurious *CDPs* detected. We have determined experimentally that a filter that eliminates all frequencies higher that 70 Hz is a good value for this parameter (see Martín et al., 2015 for more details). This process is done for all the recorded signals, so after this, we have the positions of all *CDPs* candidates for all the experiment.

After these candidates have been identified, the steps before the generation of the basic set or dictionary of events proceed as follows. The time of the maximum value within each selected signal window is considered the time-stamp of the *CDPs*. Using experts' knowledge, a suitable time window around the identified event maximum was defined. After extracting the time window, data is preprocessed in order to prepare it for clustering. Selected *CDPs* in time windows of duration *T*_*w*_ (100 ms) are resampled to reduce dimensionality from 10 to 1.6 kHz. To ease the comparison of different *CDPs*, also a potential offset is removed by subtracting the average of a subset of the initial points of the window corresponding to the beginning of the *CDP*. Given that the signal must be sufficiently smooth, *CDPs* are processed using PCA as feature extraction method to compute the most relevant dimensions that describe the whole set of identified *CDPs*. Finally, only those dimensions are used to reconstruct each *CDP*. In our experiments we use the components that explain 98% of the variance that is enough to obtain a good reconstuction of the *CDPs* and eliminates all the high frequency variations in the signal. After this step we have a collection of *CDPs* for each sensor smoothed and with their initial baseline aligned.

**Figure 2:**
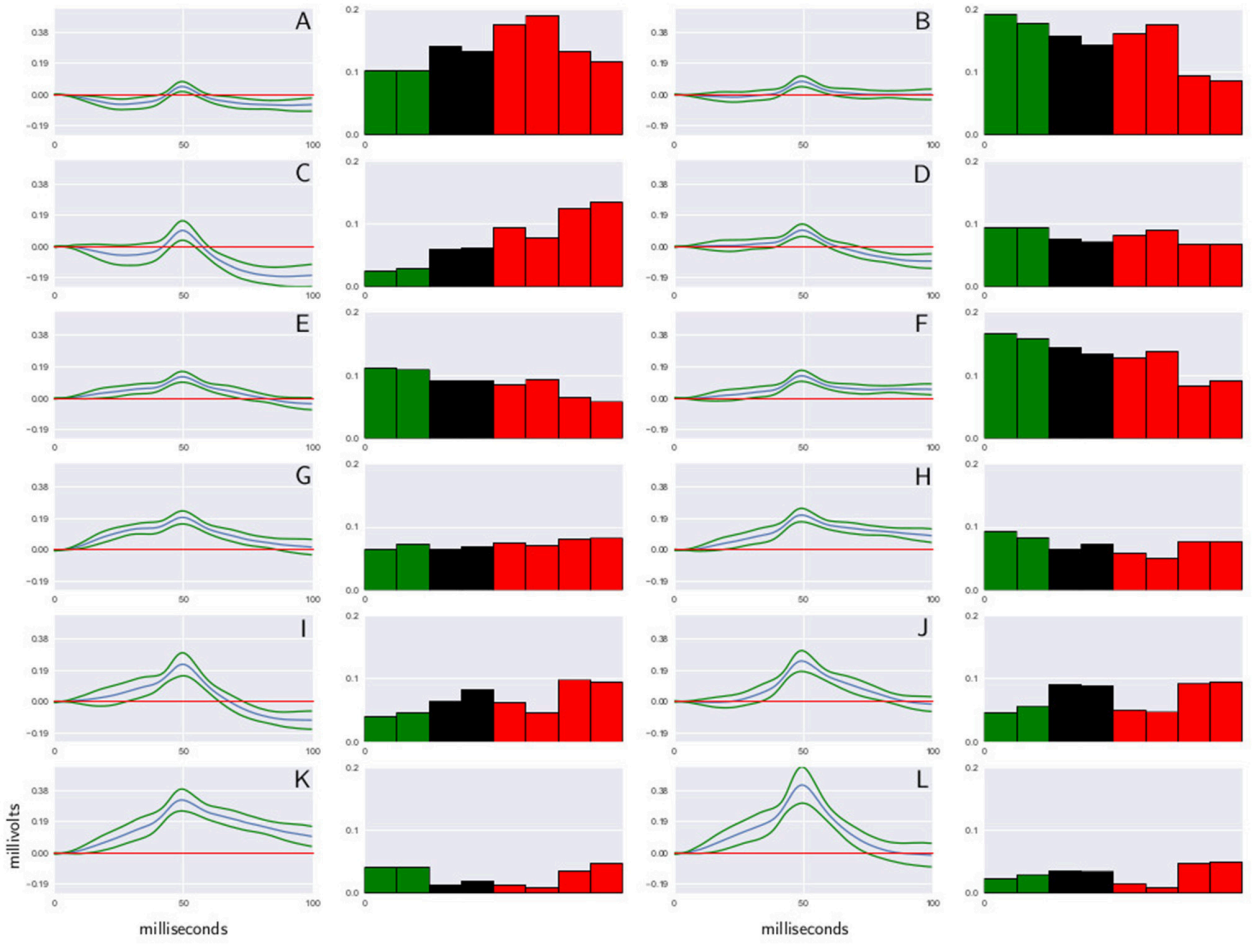
**Shape dictionary obtained from recordings made in the L6rL segment, and the probability occurrence for each shape at each time step in experiment e130221**. Each shape corresponds to the centroid of a cluster of *CDPs* and a standard deviation around the prototype. In the histograms green columns indicate the control, red columns the capsaicin, and black columns the spinalization periods. *CDPs* amplitude is measured in millivolts, time in milliseconds. Further explanations in text. **(A–L)** Represent the label assigned to each CDP class.

To build the dictionary for *CDPs* extracted from each sensor, the *k*-means algorithm is used. To select the size of the dictionaries (number of symbols), different methods for the estimation of the number of clusters are used to assure the consistency of the result. Figure 2 shows a shape dictionary obtained from potentials extracted from a specific segment in one of the experiments analyzed. This procedure was introduced in Martín et al. (2015), and shows a good performance to analyze and characterize the changes that the experimental maneuvers cause in the system.

It may be seen that this procedure allowed identification and grouping of twelve different classes of the spontaneous CDPs generated in the rostral half of the L6 segment in the left side (L6rL). This experiment was performed to examine the effects of the intradermic injection of capsaicin produced in a previously spinalized preparation. That is, in a preparation deprived of descending influences known to modulate the functional connectivity between dorsal horn neurons in response to a nociceptive stimulus. As shown by the histograms in Figure 2, the behavior of the different classes of spontaneous *CDPs* in response to the spinal section and the subsequent injection of capsaicin was not uniform: after spinalization the probabilities of occurrence were reduced in some classes of *CDPs* and increased in others. Likewise, after capsaicin the probabilities of occurrence of some classes of spontaneous *CDPs* were transiently increased and were reduced or barely affected in others. This behavior is in contrast with the pronounced changes produced by capsaicin in preparations with intact neuroaxis (e.g., experiments e110906 and e120511; see also Martín et al., 2015) and underscores the role played by the supraspinal control exerted on the capsaicin-sensitive spinal neuronal ensembles that generate the different classes of spontaneous *CDPs* (see Section 7).

As next step, the signal is discretized using symbols from the dictionary obtained for the experiment. For each sensor and step of the experiment, the sequence of extracted *CDPs* is labeled using the dictionary. The labels are assigned considering the euclidean distance to the prototypes of the corresponding dictionary. The label of the closest prototype is the label assigned to each *CDP*. A *pause symbol* ($ symbol) is introduced representing the lack of identifiable activity for a period of time. Its duration is estimated using the distribution of the time interval between consecutive *CDPs*. The mean of this distribution is taken as the duration of one pause period. Multiple pauses are included if the distance between consecutive potentials is a multiple of this estimated time. Notice that pause symbols correspond to parts of the signal not selected as *CDPs* and thus discarded. They do not correspond to periods of lack of activity in the signal. These are considered noise, random fluctuations of the signal or possible CDPs that did not have enough quality to be considered. Consequently, pauses do not have a shared shape.

**Figure 3:**
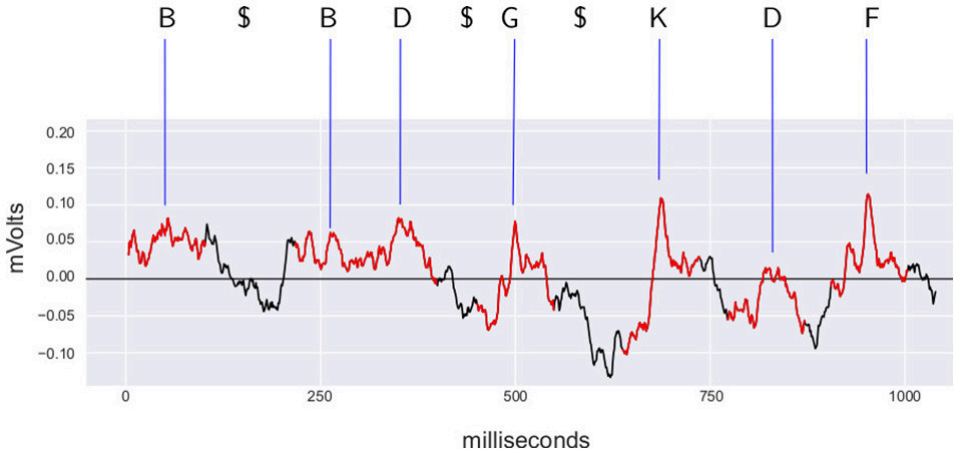
**Example of discretization of the signal recorded from L6rL from experiment e130221**. In red, the time intervals identified as spontaneous *CDPs* by the *CDP* detection algorithm used by the methodology. Each *CDP* is labeled using the shapes dictionary obtained from the segment L6rL for this experiment using the closest shape according to the euclidean distance. Symbol $ represents a *pause* in the sequence because the signal was not identified as a *CDP* (shown in black). The labels for the *CDPs* and the pause symbols form the discretized sequence. The *CDPs* in the figure correspond to the 0.3 hz–10 khz filtered signal resampled to 1.6 khz before processing with PCA.

This discretization yields a string representing the sequence of events for each segment and experimental maneuver. This process is represented in Figure 3. Typically, recordings of 10 min generate sequences of a length of 6,000 symbols at the most.

## 4. Sequential study of *CDPs*

In a previous work (Martín et al., 2015) we showed that the set of symbols in the dictionary remains the same along the whole experiment. One possibility is that the generation of particular classes of *CDPs* results from the activation of different local and relatively stable ensembles of neurons (i.e., to populations restricted to one lumbar segment). It is also possible that some classes of *CDPs* are produced by a neuronal network distributed along several lumbar segments that acquires different configurations of functionally connectivity depending on the level of neuronal synchronization, as shown in Contreras-Hernández et al. (2015). In either case, the relations between the spontaneous *CDPs* may provide significant clues on the patterns of functional connectivity between the neuronal sets involved in their generation and on how these patterns change under different experimental situations.

In this context, we will study if the firing of an ensemble of neurons (and so, the appearance of a specific *CDP*) depends on the last activated ensemble of neurons (the kind of the last appeared *CDP*). Existence of this dependence would mean that there is a minimal degree of memory in these neuronal networks, so firing of local neuronal groups is not independent from the previous activity, which may well depend on inhibitory interactions between neuronal activity as recently shown in Contreras-Hernández et al. (2015).

As a first step to study the sequential dependence among *CDPs* and its implications, the raw signal is translated into a sequence of symbols using the method already described in Section 3. Figure 3 provides an example of spontaneous *CDPs* recorded from segment L6rL during control period (*ctrl*) and its translation as a sequence of symbols. This exemplifies the kind of sequence of data that will be used for the analysis along this paper.

To study the temporal dependence between *CDPs* firing, we will start building for every pair of *CDPs* a transition probability matrix. Position row *i* and column *j* of the matrix will contain the conditional probability *P*(*c*_*j*_|*c*_*i*_) that after appearing *CDP* of kind *c*_*i*_, the next *CDP* will be of kind *c*_*j*_. There is a special column that represents the pause ($), that is, the different probabilities that after registering a *CDP*, no other detectable *CDP* will appear in 100 ms. Reciprocally, there is a special row that represents the probability that after a *pause*, another *CDP* *c*_*j*_ appears. The probability values are estimated by counting the pairs of consecutive *CDPs* appearing in the whole sequence

(1) $$P\left(c_{j}|c_{i}\right)=\frac{\text{Number of times }c_{j}\text{ is followed by }c_{i}}{\text{Number of times }c_{i}\text{ appear}}$$

**Figure 4:**
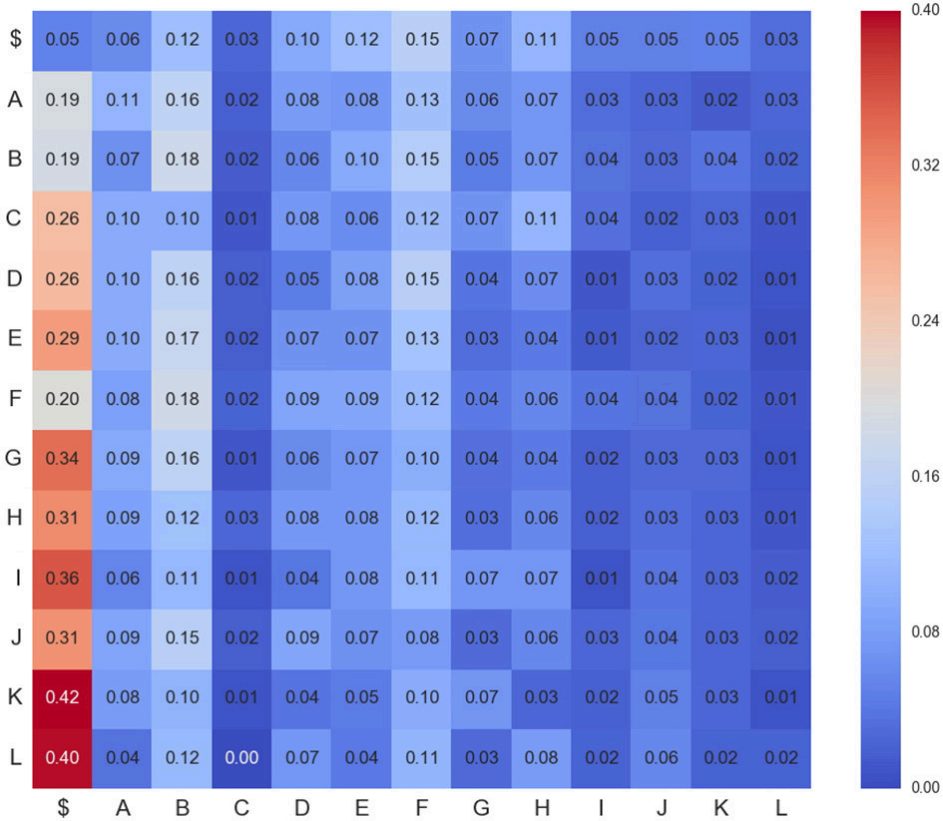
**Transition matrix for *CDPs* recorded from segment L6rL at the beginning of the experiment e130221 (*ctr*_1_)**. Position row *i*, column *j* shows probability of transition from *CDP*_*i*_ toward *CDP*_*j*_. Compare this figure with Figures 5, 6.

**Figure 5:**
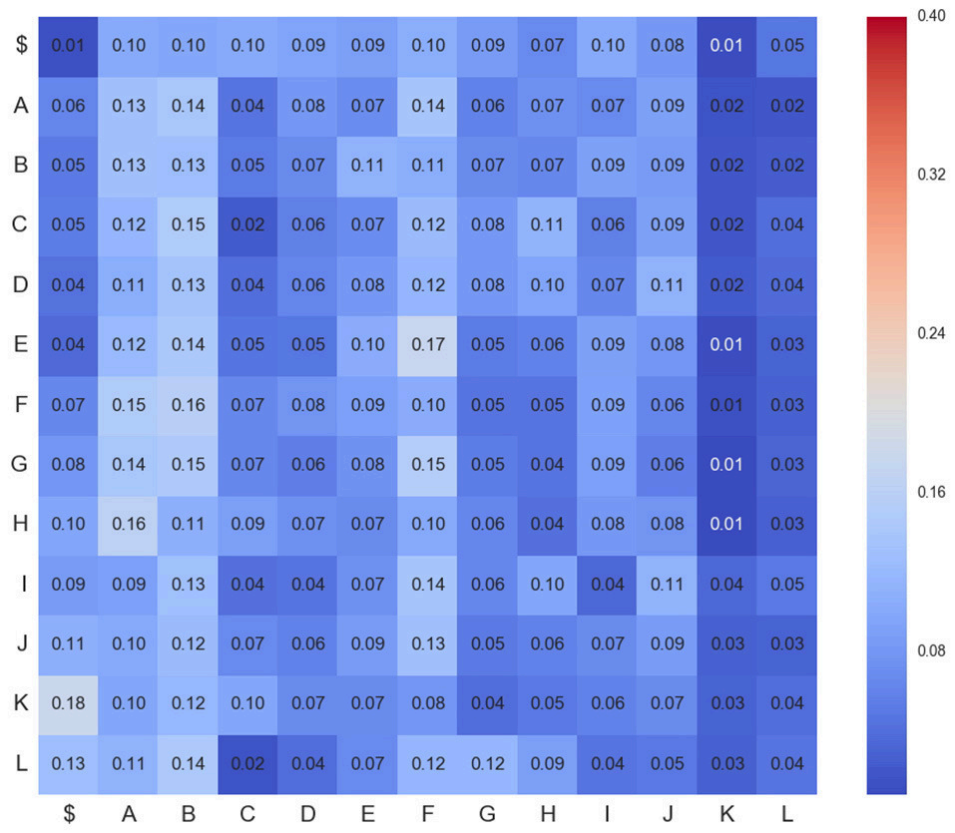
**Transition matrix for *CDPs* recorded from segment L6rL after spinalization (*esp*1)**. Same experiment and display as that of Figure 4.

**Figure 6:**
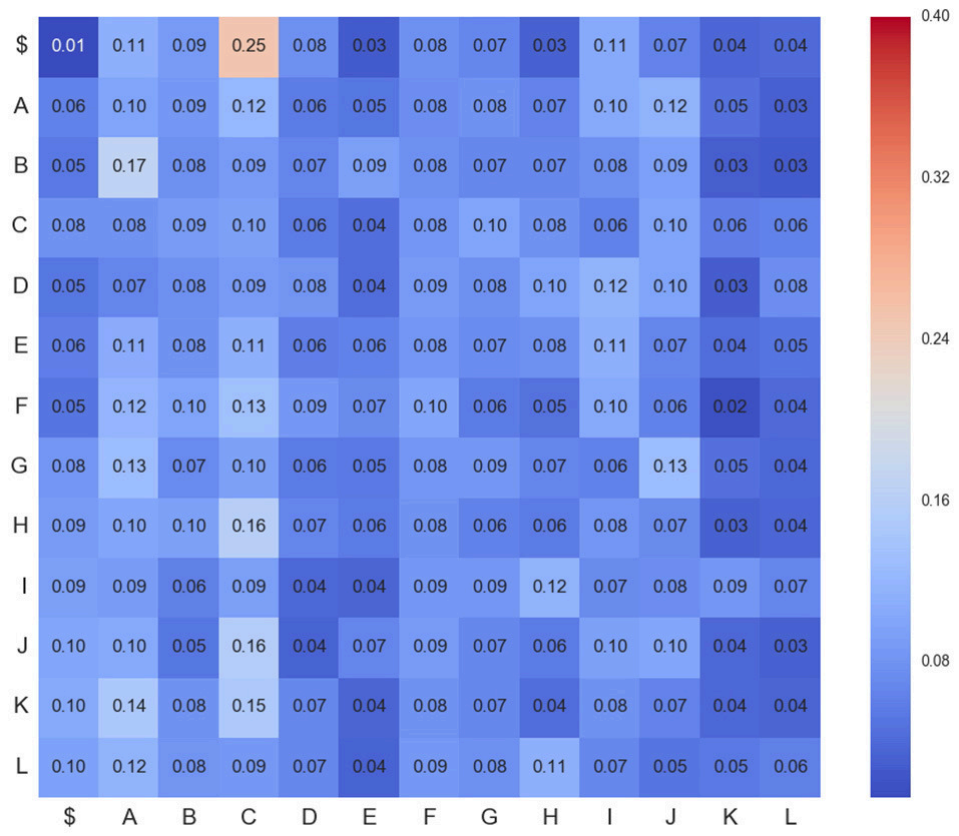
**Transition matrix for *CDPs* recorded from segment L6rL 60 min after the intradermic injection of capsaicin in the already spinalized preparation (*capsa*_3_)**. Same experiment and display as that of Figure 4.

Figures 4–**6** show three transition matrices for the same segment L6rL from different time steps of the experiment: Figure 4 in a control step, Figure 5 after spinalization, and Figure 6 after 60 min of the intradermic injection of capsaicin. A visual inspection of the matrices shows:

1. *Not all positions in a column have the same probability values*. This finding seems to support the proposal that there is a dependence between *CDPs* in time firing, because all probabilities of generating a *CDP* (all values in the column) are different depending on the previous observed one (row in that column).
2. *Visually they appear to be quite different:* So, we would expect different sequences of *CDPs* in different steps of the experiment. For instance, sequences belonging to *ctrl* would be different from those in *capsa* or *esp*. This observation suggests that it should be possible to predict from a sequence to which step of the experiment it belongs.

However, these are merely visual observations and a formal analysis of them is needed. In Section 5 we will study the first proposal and in Section 6 we will study the second one.

## 5. Markovian analysis

Time dependence between consecutive events can be formalized using *Markov chains* (Isaacson and Madsen, 1976; Florian et al., 2011). Such formalism allows studying processes with a kind of memory in which the probability of the current event depends *only* on the last *n* events occurred. Parameter *n* is called the *order* of the Markov chain and describes the degree of memory of the system. Considering the *CDPs* recorded in the sequence as events, we could model the sequence as a Markov chain of order *n* where:

(2) $$\begin{array}{r} P\left(c_{t}|c_{t-1}\ldots c_{0}\right)=P\left(c_{t}|c_{t-1}\ldots c_{t-n}\right) \end{array}$$

We will start proving that the sequence of *CDPs* recorded in all spinal segments can be modeled as a Markov chain of order one. Later we will show that the sequence is not of order higher than one.

### 5.1. First order tests

The standard way to check the markovian nature of a sequence is to perform a Pearson χ^2^ hypothesis test (Anderson and Goodman, 1957), which compares the *goodness of fit* of an observed distribution with respect to an expected distribution with *H*_0_ stating that there is no difference between them.

In our case, the test is performed by comparing the *observed* distribution of consecutive pairs appearing in the recorded sequence, with the *expected* distribution that should appear assuming independence of a *CDP* with respect to the previous one. The observed distribution consists in matrix *O* that contains in position *i, j* the number of times that *CDP* *c*_*j*_ was preceded by *CDPs* *c*_*i*_ in the recorded sequence. The expected distribution matrix *E* under the independence hypothesis stores in position *i, j* the expected number of times *CDP* *c*_*j*_ would be preceded by *CDP* *c*_*i*_ under the independence hypotheses. This number is computed as:

(3) $$\begin{array}{r} E_{i,j}=nP\left(c_{j}\right)P\left(c_{i}\right) \end{array}$$

where *P*(*c*_*j*_) and *P*(*c*_*i*_) are the frequency of *CDPs* *c*_*j*_ and *c*_*i*_ respectively in the whole recording, and *n* is the number of *CDPs* recorded. Notice that join probability *P*(*c*_*j*_, *c*_*i*_) = *P*(*c*_*j*_)*P*(*c*_*i*_) under the independence hypothesis. To translate this probability to actual expected events, we multiply this joint probability by *n*.

Hence, *H*_0_ claims that the sequence of *CDPs* recorded is generated by a renewal stochastic process and thus distribution *O* is statistically similar to *E*. *H*_0_ holding at a significant *p*-value would mean that the probability of appearing of a *CDPs* does not depend on the previous recorded *CDP*.

Once we have the observed and the expected frequencies, we compute the test-statistic

(4) $$\begin{array}{r} \chi_{n\left(n-1\right)}^{2}=\overset{n}{\underset{j,i=1}{\sum }}\frac{{\left(O_{i,j}-E_{i,j}\right)}^{2}}{E_{i,j}} \end{array}$$

that follows a χ^2^ distribution with *n*(*n*−1) degrees of freedom. This statistic measures the differences or divergence of the observed distribution O with respected to the *H*_0_-expected distribution E. The higher the value, the higher the divergence.

In the experiments shown in Table 1, for all lumbar segments and time steps of the recorded data, resulted that computed value χ^2^ was larger than expected, returning a *p*-value [following the $\chi_{n\left(n-1\right)}^{2}$ distribution]) lower than 0.0001. So *H*_0_ is extremely unlikely and, therefore, *H*_0_ must be rejected in all cases.

One problem with χ^2^ test is that it assumes that events follow a normal distribution (χ^2^ distribution is the sum of squared normal distributions), which has not been proved. So, we did a complementary test that will also be helpful later on to study possible higher orders for the Markov chain.

As in the previous case, we establish a *H*_0_ hypothesis stating that the sequence is generated by a renewal stochastic process. *H*_0_ now will be tested with a randomized test using surrogate data (van der Heyden et al., 1998). We generate 10,000 random sequences under *H*_0_ hypothesis (each sequence with the same length than the original one and definitely not markovian), keeping the frequency of each *CDP* equal to that of the original sequence. Each sequence is simply generated as a *random permutation* of the original sequence, so each one maintains the frequency of each *CDP* but any markovian structure is removed because the previous *CDP* is randomly changed.

If the original sequence is not markovian, it is expected to find that the divergence of *O* with respect to *E* computed using Equation (4) is similar to the divergence of probability matrices obtained from surrogate data to *E*. So, we computed for each of the 10,000 randomized sequences the divergence in their distribution with respect to the expected matrix.

Results showed that all randomized sequences had divergence smaller than the original sequence. We could expect some of the randomized sequences to have, by chance, a smaller divergence value than the original one. The number of such sequences would determine the *p*-value for hypothesis *H*_0_. None of them had a higher deviation, so we conclude that the *p*-value for *H*_0_ is lower than 1 over 10,000. Thus, as expected, *H*_0_ is again rejected.

### 5.2. Higher orders tests

We also tried to test if the markovian process is of order two. That would mean that appearance of one *CDP* depends on the last two preceding *CDPs*. Given that we know that the sequence is at least of order one, now hypothesis *H*_0_ is that the sequence is not of order two.

In this case, building a χ^2^ test is difficult as many of the entries in the observation matrix become zero. Notice that now transition matrices are from pairs of *CDPs* to a single *CDP*, so the size of matrix *O* is *n*^3^. For our experiment, with 13 symbols we have 2.197 entries in the matrix. With sequences of at the most 6,000 symbols we can not compute a reliable statistic. This, added to the hypothesis of normal distribution, motivated us to use a randomized test. Again, we had to generate randomized sequences following *H*_0_, that is, the frequency of each pair of consecutive *CDPs* is maintained as in the original sequence, but removing possible second order dependencies, thus removing existing dependencies among triplets of consecutive *CDPs*. This randomization can be done following the Whittle's algorithm (Pethel and Hahs, 2014). We reduced the number of randomized sequences from 10,000 to 1,000. With that number of surrogate examples, we have enough power to estimate a significant *p*-value of 0.001.

In all experiments described in the paper, we had an average *p*-value of 0.34, which is not enough to reject *H*_0_, so we must conclude that most probably the sequences are not of order two.

We don't have sufficient data to produce a statistically significant test for higher orders of dependence, so conclusion should be that sequences of *CDPs* cannot be explained by a renewal stochastic process and that they should be modeled by a markov process at least of order 1.

## 6. Predicting sequences

Once we know that *CDPs* sequences are markovian of order one, we wonder if we can take advantage of this effect to *recognize*, given a short sequence of raw recording, to which step of the experiment it belongs.

We explore this possibility by building a transition matrix (*m*_*l, s*_) for each lumbar segment *l* in step *s* of the experiment. This matrix represents our model of the sequence. Such models consist in the set of transition probabilities between each pair of *CDPs*:

(5) $$\begin{array}{r} m_{l,s}=\left\{P\left(c_{l,s}^{t+1}=c_{i}|c_{l,s}^{t}=c_{j}\right)|\forall c_{i},c_{j}\in \text{CDPs}\right\} \end{array}$$

where probabilities of transitions *P*(*c*_*i*_|*c*_*j*_) are estimated from the sequence **C**_*l, s*_ of *CDPs* recorded in lumbar segment *l* and step *s*. This set of probabilities corresponds to the observed transition matrix, used in previous markovian tests and depicted in Figures 4, 6. Table 2 contains a description of the notation.

**Table 2:** **Description of major symbols**.

**Notation**	**Description**
L	Set of lumbar segment
*s*	Time step, a contiguous subset of the recording
S	Set of time steps
$C_{l,s}$	Sequence of **CDPs** from lumbar segment *l* and time step *s*
$C_{l,s}^{k}$	Subsequence of the last *k* **CDPs** from sequence $C_{l,s}$
$C_{l,s}^{n-k}$	Subsequence of **CDPs** from sequence $C_{l,s}$ without the last *k* **CDPs**
$c_{l,s}^{t}$	CDP from a sequence $C_{l,s}$ at time instant *t*
*m*_*l, s*_	Probability model for lumbar segment *l* and time step *s* estimated from a sequence $C_{l,s}$ of **CDPs**
$m_{l,s}^{n-k}$	Probability model for lumbar segment *l* and time step *s* estimated from sequence $C_{l,s}^{n-k}$
$P\left(C_{l,s}|m_{l,s}\right)$	Likelihood of sequence $C_{l,s}$ for lumbar segment *l* and time step *s* given model *m*_*l, s*_
$P\left(c_{l,s}^{t+1}|c_{l,s}^{t};m_{l,s}\right)$	Probability of **CDP** $c_{l,s}^{t+1}$ given **CDP** $c_{l,s}^{t}$ and model *m*_*l, s*_
*S*(*s*_*i*_, *s*_*j*_)	Similarity index between time step *s*_*i*_ and *s*_*j*_

The complete experiment is modeled by set M:

(6) $$\begin{array}{r} M=\left\{m_{l,s}|\forall l\in L,s\in S\right\} \end{array}$$

where L is the set of studied lumbar segments, and S is the set of recorded temporal steps. Remember that a temporal step consist in data recorded that spans for several minutes, usually 10, during the experiment.

The Markovian property of conditional dependence limited to only the last *CDP*, allows us to easily compute from a model *m*_*l*,_*s*__*i*__ the *likelihood* to generate data **C**_*l*,_*s*__*j*__ recorded in another time step *s*_*j*_.

Given that we have a markov process of order one, the probability for a *CDP* *c*^*t*+1^ depends only on the previous *CDP* *c*^*t*^, so for a lumbar segment *l* and time step *s*_*i*_ and model *m*_*l*,_*s*__*i*__ its probability can be defined as:

$$P\left(c_{l,s_{j}}^{t+1}|c_{l,s_{j}}^{t};m_{l,s_{i}}\right)$$

For a sequence of *CDPs* **C**_*l*,_*s*__*j*__, the probability of each individual *CDP* is dependent only on the previous *CDP*, and so independent of the other ones. In this case, the probability of the sequence **P**(**C**_*l*,_*s*__*j*__|*m*_*l*,_*s*__*i*__) can be factorized as the product of the individual conditional probabilities:

(7) $$\begin{array}{r} \text{P}\left({\text{C}}_{l,s_{j}}|m_{l,s_{i}}\right)=\overset{n-1}{\underset{t=1}{\prod }}P\left(c_{l,s_{j}}^{t+1}|c_{l,s_{j}}^{t};m_{l,s_{i}}\right) \end{array}$$

where **P**(**C**_*l*,_*s*__*j*__|*m*_*l*,_*s*__*i*__) is the probability for model *m*_*l*,_*s*__*i*__ to generate sequence **C**_*l*,_*s*__*j*__, *n* is the length of sequence **C**_*l*,_*s*__*j*__, and $P\left(c_{l,s_{j}}^{t+1}|c_{l,s_{j}}^{t};m_{l,s_{i}}\right)$ is the probability in model *m*_*l*,_*s*__*i*__ of occurring $c_{l,s_{j}}^{t+1}$ in position *t*+1 of sequence **C**_*l*,_*s*__*j*__ *given* that in position *t* of the same sequence appeared $c_{l,s_{j}}^{t}$.

Instead of working with probability values, we will work (for numerical stability reasons) with the logarithm of the probabilities:

(8) $$\begin{array}{rc} log\left(\text{P}\left({\text{C}}_{l,s_{j}}|m_{l,s_{i}}\right)\right) & =log\left(\overset{n-1}{\underset{t=1}{\prod }}P\left(c_{l,s_{j}}^{t+1}|c_{l,s_{j}}^{t};m_{l,s_{i}}\right)\right) \end{array}$$

(9) $$\begin{array}{r} =\overset{n-1}{\underset{t=1}{\sum }}log\left(P\left(c_{l,s_{j}}^{t+1}|c_{l,s_{j}}^{t};m_{l,s_{i}}\right)\right) \end{array}$$

### 6.1. Experiments

**Figure 7:**
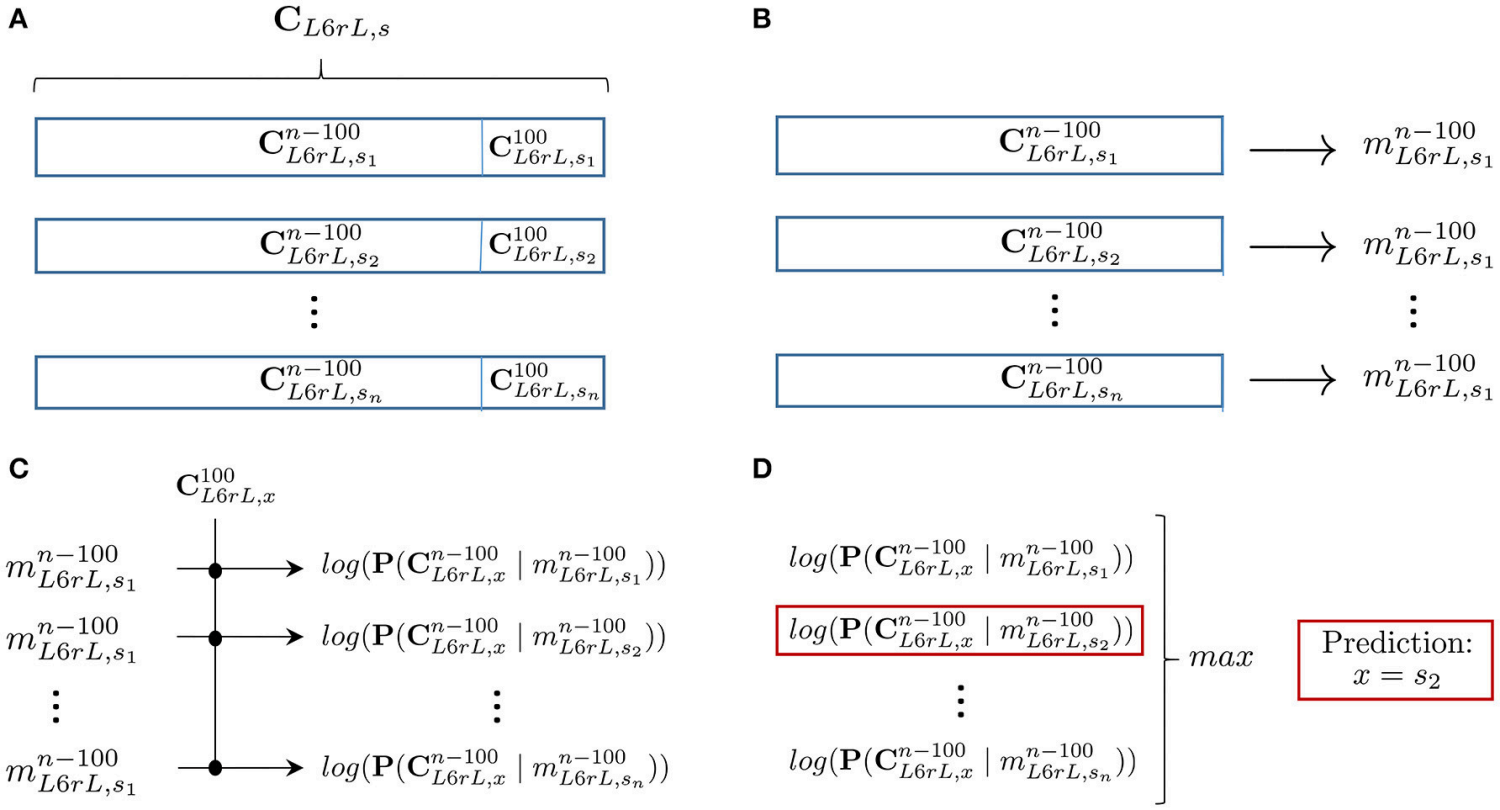
**Summary of the prediction process described in Section 6.1 for segment (*L*6*rL*): (A)** For each 10 min step, we remove the last 100 *CDPs* of the recorded sequence ($C_{l,s}^{100}$). **(B)** Building, for each step of the experiment, the model with the remaining data ($C_{l,s}^{n-100}$). **(C)** Given an unknown sequence of 100 *CDPs*, computation of the log-likelihood of each model for that particular sequence. **(D)** Obtaining the model with maximum log-likelihood and return prediction of membership. Average prediction accuracy for all segments in several experiments is shown in Table 3.

To test if likelihood can help to recognize for a sequence of recorded data the type of time step it belongs to, we performed the following experiment (see Figure 7): for each lumbar segment *l* and time step *s*, we extracted sequences consisting of its last 100 *CDPs*. This corresponds roughly to 12 s of recorded data. We call this sequences ${\text{C}}_{l,s}^{100}$. The task will be to recognize the source sequence from which a sequence of 100 *CDPs* was extracted. Using the remaining data in the sequence (${\text{C}}_{l,s}^{n-100}$), we built a model $m_{l,s}^{n-100}$. Notice that the probabilities are now computed using a shorter sequence of data (all data recorded except the last 100 *CDPs*). This is done with the purpose of implementing a cross-validation procedure, avoiding the use of the testing sequence to build the model. After this procedure is performed, we have for each step *s* and lumbar segment *l*, a sequence of 100 *CDPs* (${\text{C}}_{l,s}^{100}$) and a model $m_{l,s}^{n-100}$.

Subsequently: for a given lumbar segment *l*, we randomly selected a sequence ${\text{C}}_{l,*}^{100}$ of last 100 *CDPs* of unknown origin. The task consisted in trying to predict the time step *s* from which it was extracted. In order to do so we computed, using equation 8, the likelihood of each model $m_{l,s}^{n-100}$ to generate ${\text{C}}_{l,*}^{100}$. The model with higher likelihood should belong to the time step that originated the sequence. However, notice that there may be several recordings, each one 10 min long, for each step of the experiment.

**Table 3:** **Average percentage of success in membership prediction for all experiments with respect to the length of the sequence**.

**Experiment**	**Random classifier**	**Likelihood classifier Length of sequence to predict**					
		**50**	**100**	**150**	**200**	**250**	**300**
e110906	51.9	85	92.5	95	92.5	92.5	95
e120511	42.8	58.4	65.0	68.8	71.4	63.6	68.8
e130221	37.4	80.7	88.6	88.6	92.0	91	93.2
e140225	37.4	76.4	75.0	77.7	75.0	73	77.1

*In general, the longer the sequence used to predict, the better the results obtained. First column shows average accuracy obtained from a random classifier*.

For instance, we have in experiment e130221 two control recordings, two more spinalization recordings and, finally, four capsaicin recordings. Trying to predict for that experiment where the sequence of the last 100 *CDPs* of sensor *L*6*rL* of *ctrl*_1_ (${\text{C}}_{L6rL,ctrl_{1}}^{100}$) belongs, returns the following log-likelihood values for each model:

Likelihood for model *ctrl*_1_: -217.56
Likelihood for model *ctrl*_2_: -216.50
Likelihood for model *esp*_1_: -253.80
Likelihood for model *esp*_2_: -247.84
Likelihood for model *capsa*_1_: -252.60
Likelihood for model *capsa*_2_: -360.21
Likelihood for model *capsa*_3_: -261.66
Likelihood for model *capsa*_4_: -267.26

Notice that log-likelihood values are always negative (given that probabilities are lower or equal than 1) and that the higher the value of the log-likelihood is, the higher the likelihood. We will consider a success when the predicted sequence belongs to the right time step. In the example shown above, the most likely model to generate the sequence is *ctrl*_2_, because it has the highest log-likelihood. We take it a success in prediction because it corresponds to a control step recording.

This procedure has been repeated for all testing sequences ${\text{C}}_{l,s}^{100}$ of each state *s* for all lumbar segment *l*, and success in prediction was averaged. For instance, in experiment e130221, the average of success in prediction is 88.6%. A random classifier would have a success rate of 37.4% in this case.

Table 3 shows average prediction accuracy among all segments and time steps in all the considered experiments. It also shows how accuracy changes as the length of the sequence used to predict also varies. For larger sequences the success rate is higher. For comparison, the table also shows accuracy of a random classifier for each experiment. Each one has a different result for the random classifier because each experiment has a different number of time steps for each kind (see Table 1). Values for the random classifier have been obtained through a standard Montecarlo estimation.

### 6.2. Results

Notice that predictions are not always 100% *successful*. However, instead of considering that as an error, we think that it also could explain something about the inner structure of the spinal neuronal connectivity.

For instance, in experiment e110906 100% accuracy is consistently achieved in all segments except in segments L6rR, L5rR, L5cR, all of them from the right side of the cat's spinal cord. In all experiments, the injection of capsaicin was applied in the left hindlimb, and this explains the *different* effect in the right side of the spinal cord (see also Figure 1).

Nevertheless, though prediction results of a particular recording of a given time step for a lumbar segment could not be always accurate, the consensus of all lumbar segments it is very accurate as shown in Table 4 that provides a detailed view of results for experiment e130221. Notice that considering the most common prediction for all lumbar segments in a given time step, results are 100% correct in all experiments (Rokach, 2010).

**Table 4:** **This table details the prediction results in experiment e130221**.

	***ctrl*****_1_**	***ctrl*****_2_**	***esp*****_1_**	***esp*****_2_**	***capsa*****_1_**	***capsa*****_2_**	***capsa*****_3_**	***capsa*****_4_**
L4cR								
L4cL								
L5cR								
L5cL								
L5rR								
L5rL								
L6cR								
L6cL								
L6rR								
L6rL								
L7rL								
Consensus								

*Each cell shows prediction for $C_{l,s}^{\text{100}}$, where l is the spinal segment (row) and s is the time step (column). Prediction is represented in colors: green means that prediction belongs to a ctrl step, black means that prediction is a esp step, and red means a capsa step. In this case, ideally, the two first columns should be green, next two black, and at the end four columns in red. The number of cells properly colored with respect to the total number of cells corresponds to accuracy reported in Table 3. Last row labeled as Consensus represents the most voted prediction for all rows in that column. Notice that in this and all experiments, voting consistently provides the proper prediction*.

Finally, by examining the errors in prediction, we can see that in most cases the right prediction has the second highest log-likelihood, and it is very close to the highest one. This can be observed in Table 5 where we change the successful prediction criteria to have the right prediction among the *k* models with higher log-likelihood. Notice how precision is increased as we are more permissive in this criteria.

**Table 5:** **Percentage of success defined as proper prediction among the set of *k* models with higher log-likelihood**.

**Experiment**	***k***			
	**1**	**2**	**3**	**4**
e110906	92	95	100	100
e120511	65	86	94	94
e130221	89	95	99	100
e140225	75	88	97	100

These results suggest that the likelihood approach used allows a valid prediction of the step where data were originated, even for small sequences of data. It also encourages us to try to build a similarity index between different steps of the experiment in order to understand the high level behavior of the neural networks during an experiment.

## 7. Discussion

### 7.1. About the method

In this paper we used data from four experiments (see Table 1) to identify a class of Markov processes that describe the local interactions between the populations of dorsal horn neurones which generate the spontaneous *CDPs* examined by Contreras-Hernández et al. (2015) and Béjar et al. (2015).

We found that in these experiments the spontaneous *CDPs* recorded from a particular spinal segment are markovian and have order one, as shown in Section 5. That is, the generation of a particular type of *CDP* depends on the type of the preceding *CDP*. In addition, we also found that this markovian property can be used to build a procedure to identify portions of signal as belonging to a specific functional state of the connectivity between the neuronal networks involved in the generation of the *CDPs*. The functional implications of these findings are discussed in Section 7.2. Although, our results agree with the findings reported by Contreras-Hernández et al. (2015), the functional organization of the ensembles of neurons that produce this information has to be further investigated. To this end, and based on the idea that the studied processes are markovian (see Section 6), we introduced a method to predict the membership of a sequence ${\text{C}}_{l,*}^{100}$ of the last 100 *CDPs* of a time step reds∈S (see Figure 7). In Table 3, we show the accuracy of this method applied to the four selected experiments to validate the hypothesis.

The log-likelihood of a subsequence given a model can also be used as a similarity criterion among time steps (*s* ∈ *S*). This allows to partition the time steps according to their similarity and to define a set of classes characterizing the different behaviors during an experiment. These classes are still to be characterized and further studied. We believe that this analysis will contribute to understand *how* the patterns produced by ensembles of neurons change during different experimental situations such as nociceptive stimulation (*capsa*) or acute spinal lesions (*esp*).

Some concerns could arise about the impact of the preprocessing of raw signal on the obtained results. Specifically there are few parameters that could impact on the extracted sequences used to test the markov properties of the signal. First of all, for the extraction of the CDPs we work on the raw signal (at 10 kHz frequency) filtering frequency only to detect the position of the signal maximum in the selected windows. Filtering involves the elimination of frequencies higher than 70 Hz, enough to filter out most of the noise. In Martín et al. (2015) we provide details about the effect of different frequency cutoffs and the relative signal-to-noise ratio.

Another parameter affecting the sequences is represented by the data denoising. To this end PCA is performed allowing to reconstruct signals using PCA components accounting up to 98% of the variance. As a result, a large portion of the high frequency noise is eliminated without significantly compromising the shapes of the selected CDPs.

Finally, last parameter affecting the sequences selection is represented by the number of clusters used to compute the symbols dictionary. We implemented an optimal methodology for selecting the best suited number of clusters for the set of CDPs (Martín et al., 2015). The proposed methodology generates the optimal number of clusters also yielding other suboptimals with smaller or larger number of clusters. Using these subobtimal could in principle affect the markovian properties of the signal. However, to prove that even in these cases the markovian property still holds, we did some experiments with the same raw data but using a suboptimal number of clusters for building the *CDPs* dictionaries. In particular, we applied the randomized methodology described in Section 5.1 to test whether sequences show the markovian property of order 1. The *p*-values obtained for each experiment are reported in Table 6. Eventually each p-value shown is the maximum obtained in all sequences (with different segment and step) of the experiment. The results show that even in those cases the significance of the markov property is high.

**Table 6:** **Obtained *p*-values testing markov behavior of degree 1 for each experiment varying the number of symbols considered in raw signal**.

**Experiment**	**Optimal**	**8**	**15**
e110906	5.30e-25	1.25e-07	1.07e-34
e120511	1.72e-34	1.99e-54	1.72e-34
e130221	6.83e-30	3.62e-38	6.83e-30
e140225	3.54e-23	4.79e-50	3.54e-23

*Each value presented is the maximum p-value obtained for all sequences of the experiment. In all cases we have a p-value a lot lower than 0.05 that show robustness of the detection of the markovian property with respect to the number of symbols considered*.

In conclusion, we can see that the detection of the phenomena is robust when changing the parameters of the pre-processing steps of the methodology. Notice also that the effect of introducing false CDPs in our analysis, given that noise is not markovian, would only add difficulty in detecting the markovian property. However, our analysis shows clearly, with a *p*-value very close to zero for all experiments, that the markovian property of degree at least one is fullfilled by all the sequences studied.

### 7.2. Some functional implications

Previous studies in the spinal cord of the anesthetized cat have been addressed to examine the functional organization of the dorsal horn neurons contributing to the generation of the spontaneous negative and negative-positive *CDPs* (*nCDPs* and *npCDPs*, respectively). During low levels of dorsal horn neuronal synchronization, the network would generate *nCDPs* and activate the pathways mediating non reciprocal post-synaptic inhibition. In contrast, during high levels of neuronal synchronization, *npCDPs* would be mostly generated and would preferentially activate the pathways mediating presynaptic inhibition.

These studies showed in addition that the spontaneous *nCDPs* and *npCDPs* were followed by a silent phase during which no other spontaneous *CDPs* were generated. Since the *CDPs* evoked by stimulation of a cutaneous nerve were also depressed when preceded by spontaneous *nCDPs* and *npCDPs*, it was concluded that the silent phase was due to the concurrent activation of GABAergic and glycinergic inhibitory pathways (Chávez et al., 2012; Contreras-Hernández et al., 2015). Yet, the analytic methods available at that time provided limited information on the functional organization and possible selectivity of these inhibitory interactions.

We now used Markov processes to describe the interactions between spontaneous *CDPs* recorded in a particular spinal segment. As shown in Section 5, we found in all experiments that the generation of the *CDPs* could be described by a Markov order one process, which means that the network had memory and that generation of a particular kind of *CDP* depended on which *CDP* was previously generated. We also found that these interactions were non-random, a situation consistent with the proposal that individual *CDPs* are followed by a structured activation of a segmentally distributed and rather selective inhibitory networks that contribute to the shaping of the sensory information arriving from the periphery.

The main outcome of the markovian analysis was the finding that the time sequence of the spontaneous *CDPs* extracted from the potentials recorded from a given segment was also non-random and had a specific structure that could be associated with a particular functional state of the network. That is, the temporal sequences of the spontaneous *CDPs* obtained from control (*ctrl*) recordings were basically different from those sequences recorded after spinalization (*esp*) that is on spinal segments devoid of supraspinal control or after capsaicin (*capsa*). We would be dealing here with a signature like process that characterizes the configuration of the neuronal connectivity of the whole neuronal ensemble during a particular functional state.

From a functional point of view, it is clear that in a chain of in series interconnected sets of neurones the activation of each set will affect the responses of the next set in the chain and so on a situation that could lead to Markov processes of different orders. In the case of the spontaneous *CDPs* presently analyzed we are dealing with ensembles of mutually interconnected sets, where each set is influenced by the activity of the other sets. Therefore, it is quite feasible that the effects of a particular class of *CDP* on the subsequent *CDP* will be probably stronger than interaction with more remote events.

Although, discussion on the functional implications of these findings is beyond the scope of this publication, it should be noted that at present time we have limited information pertaining the kind of dorsal horn neurons involved in the generation of the different classes of CDPs except for the negative and negative positive *CDPs* (see Contreras-Hernández et al., 2015) where it was shown that depending on the magnitude of the correlation between the neurons the same ensemble may generated *nCDPs* and *npCDPs*. So the question remains on whether the same set of neurons generates the different classes of *CDPs* or whether each of them is generated by a specific set of tightly coupled neurons. Nevertheless, it is tempting to suggest that the segmentally correlated sequences of neuronal activity might be acting as a dynamic functional switch, directing the flow of sensory information arriving to the spinal cord. They might be acting by introducing, via presynaptic mechanisms, a specific signature to the ensemble of sensory fibers to inform the supraspinal structures on the *functional state* of the spinal cord, which may in turn modulate the state of neuronal sensitization induced by nociception.

### 7.3. Relation with other previous works

Our observations indicate that *CDP* sequences cannot be generated by a renewal (memoryless, or Markov order zero) stochastic process, but rather by a Markov order one stochastic process. Other previous works in neuronal activity also found temporal dependences in neuron firing. For instance, Ratnam and Nelson (2000), based on the analysis of the time sequences of the action potentials transmitted by single sensory fibers of the electric organs of the *Apteronotus leptorhynchus*, concluded that a Markov process of at least fourth-order is required to adequately describe spike activity.

This could seem to be in contradiction with our work. Yet it must be pointed out that this situation is completely different from the *CDPs* presently analyzed because they are produced by populations of interconnected neurons whose activity is determined, in addition to the intrinsic patterns of neuronal connectivity, and by excitatory and inhibitory influences generated at segmental and supraspinal level.

As demonstrated in a preceding paper (Contreras-Hernández et al., 2015), the *CDPs* are followed by an inhibitory period attributed to the activation of GABAergic and glycinergic pathways. The duration of this inhibitory phase is quite variable and we believe it plays a significant role in setting up the probabilities of occurrence of the next *CDP*. This by itself could impose a non-renewal Markov one process in the network. It is clear to us that the preceding events could also exert some influence on the final outcome. But in this sense we must keep in mind that we are dealing here with networks of interconnected neurons where it is really difficult (but not impossible) to establish causal relations. We believe that our inability of detect higher order Markovian processes is more a consequence of the properties of these networks rather than a limitation of the employed computational procedures.

### 7.4. Future work

At this point, two lines of research emerge from this work.

First, we have seen that our method is able to predict the maneuver from which a data sequence was registered (see Section 6). However, our method can introduce a bias in some cases. For instance, it may happen that the behavior of sequences from different maneuvers could correspond to the same functional state of the spinal cord. An example could be when, after some time, the effect of capsaicin injection disappears and the state of the spinal cord returns to something similar to control, or when the application of some drug cancels the effect of capsaicin. In those cases time steps belonging to different maneuvers can be confused. We plan to extend this study in an unsupervised manner so the method could be able to detect changes in behavior of the state not defined by the label of last maneuver made on the cat.

Second, we would like to study the role of synchronization of events across all the lumbar segments. The objective of this study will be to enhance the model of the sequential behavior and to cast some light about the functional connectivity among different neural groups generating *CDPs* in different lumbar segments. This analysis is complementary to the presented here. Markovian analysis could be considered as a horizontal analysis (considering time dimension in the *x*-axis) of temporal dependences. Study of synchronizations could be considered a vertical study (synchronization in the same time step) of the signals at the same time. However, for this other study, the markovian analysis does not help because we lose the temporal dimension.

## Author contributions

DC, EC, SG, and PR from the “Centro de Investigaciones y Estudios Avanzados” in Mexico, together with the help of SG, designed and performed the experiments described in the paper. MM, JB, GE, and UC from the “Universitat Politécnica de Catalunya” suggested and performed the Markovian analysis of the experimental data. All of them helped in the interpretation of the results and conclusions.

## Ethics statement

Cats were bred and housed under veterinary supervision at the Centro de Investigaciones y Estudios Avanzados of the Instituto Politécnico Nacional Animal Care unit (SAGARPA permission AUT-B-C-0114-007). All experiments were approved by the Institutional Ethics Committee for Animal Research (protocol no. 126-03) and of the National Institutes of Health (Bethesda, MD, USA; Animal Welfare Assurance no. A5036-01). The Guide for the Care and Use of Laboratory Animals NRC (2010) was followed in all cases.

### Conflict of interest statement

The authors declare that the research was conducted in the absence of any commercial or financial relationships that could be construed as a potential conflict of interest.
